# Supplementary material for: The Gut Microbiome among Postmenopausal Latvian Women in Relation to Dietary Habits
Source: Nutrients. 2022 Aug 30;14(17):3568. doi: 10.3390/nu14173568 (PMC9460340; doi:10.3390/nu14173568)
Supplement: Supplementary file 1 [file nutrients-14-03568-s001.zip › nutrients-1784541-supplementary.pdf]

Supplementary materials:

Table S1. Categories of food products and beverages.

Category	Examples of products from the specific category
Grains, grain-based products	Pasta, oats, rice, breakfast cereals, muesli, etc.
Bread	White bread, rye bread, seed bread
Potatoes	Boiled, fried potatoes, French fries
Meat, offal	Pork, beef, lamb, poultry, liver
Meat products	Sausages, smoked meat, canned meat
Eggs	Hen eggs, quail eggs
Fish	Freshwater fish, seawater fish
Fish products	Canned fish, fish sticks, smoked fish
Milk and milk products	Milk, kefir, buttermilk, yoghurt, sour cream, cottage cheese, cheese, etc.
Vegetable oils, plant and animal-based fats	Olive oil, other vegetable oils, margarine, butter, etc.
Vegetables	Fresh and cooked vegetables
Fruits, berries	Fresh and dried fruits, fresh berries
Mushrooms	Wild and cultivated edible mushrooms
Legumes, nuts, seeds, milk alternatives	Beans, peas, lentils, soy and its products, nuts, peanuts and peanut butter, plant-based drinks, tofu, vegan cheese, etc.
Sweets, bakery goods	Pastries, cookies, chocolate and other candies, etc.
Condiments	Sugar, honey, sugar substitutes, soy sauce, mayonnaise
Fast food, salty snacks	Hamburgers, pizza, dumplings, instant soups, potato and corn chips
Lemonades, energy drinks	Lemonades with and without caffeine and sugar, energy drinks
Coffee	Filtered coffee, instant coffee, etc.
Tea	Black tea, green tea, herbal tea, etc.
Water	Tap water
Dietary supplements	Fish oil, multivitamins, iron, calcium, vitamin D, vitamin C, magnesium, B group vitamins, probiotics, fibre supplements, meal replacements, other dietary supplements

Table S2. Calculated average daily intake (g per day) [1] of food products and beverages based on data from the food frequency questionnaire (n = 52).

Food products or beverages	Control group (n = 26)	Experimental groups (n = 26)
Grains, grain-based products	82.11 ± 101.77 (3.66–482.22)	65.87 ± 65.54 (1.74–313.78)
Bread	11.34 ± 9.38 (0.27–31.06)	12.20 ± 10.36 (0.00–40.79)
Potatoes	38.38 ± 31.70 (3.95–120.00)	54.36 ± 38.40 (0.00–147.78)
Meat, offal	63.00 ± 50.15 (0.00–230.55)	81.83 ± 49.93 (0.00–198.58)
Meat products	7.81 ± 8.96 (0.00–31.66)	14.48 ± 15.60 (0.00–70.52)
Eggs	29.53 ± 31.19 (1.97–150.33)	27.27 ± 15.27 (4.93–60.08)
Fish	31.98 ± 39.56 (0.82–200.00)	28.77 ± 31.27 (3.56–156.71)
Fish products	9.25 ± 15.69 (0.22–68.55)	7.05 ± 12.21 (0.25–62.68)
Milk and milk products	320.78 ± 245.75 (11.84–875.62)	344.08 ± 188.47 (0.00–824.52)
Vegetable oils, plant- and animal-based fats	14.65 ± 11.91 (1.38–54.87)	11.82 ± 5.49 (1.49–25.61)
Vegetables	124.00 ± 64.00 (23.67–280.00)	177.62 ± 138.69 (39.89–700.00)
Fruits, berries	170.96 ± 150.54 (12.82–525.00)	158.31 ± 125.77 (22.93–464.96)
Mushrooms	2.17 ± 2.24 (0.08–6.41)	1.31 ± 1.70 (0.00–6.41)
Legumes, nuts, seeds, milk alternatives	35.36 ± 37.37 (2.38–144.05)	32.86 ± 35.96 (0.25–162.96)
Sweets, bakery goods	15.45 ± 15.41 (0.00–59.18)	21.78 ± 21.09 (0.00–67.32)
Condiments	6.54 ± 7.12 (0.00–23.63)	6.82 ± 5.65 (0.00–20.62)
Fast food, salty snacks	14.92 ± 23.34 (0.00–104.49)	11.12 ± 16.15 (0.44–71.12)
Lemonades, energy drinks	15.70 ± 25.99 (0.00–110.30)	29.32 ± 51.10 (0.00–220.00)
Coffee	313.57 ± 267.07 (0.00–1080.00)	445.68 ± 228.70 (119.67–1080.00)
Tea	260.75 ± 320.18 (0.00–1440.00)	270.08 ± 219.77 (1.97–600.00)
Water	877.58 ± 398.64 (47.01–1320.00)	941.63 ± 417.55 (172.38–1320.00)

[1] Mean ± standard deviation (minimal–maximal value).

Table S3. Significant correlations between food, beverage intake and gut microbiome.

Food products or beverages	Taxonomic gut microbiome composition	Spearman's rank correlation coefficient (ϱ), *p*-value
Grains, grain-based products	genus *Bifidobacterium*	ϱ = 0.517, p = 0.002
	genus *Atopobium*	ϱ = 0.513, p = 0.003
Potatoes	*Enterobacter* spp.	ϱ = -0.532, p = 0.002
Meat, offal	genus *Gastranaerophilales*	ϱ = -0.500, p = 0.004
	genus *Victivallis*	ϱ = -0.600, p < 0.0001
	genus *Anaeroplasma*	ϱ = -0.521, p = 0.002
	genus *Terrisporobacter*	ϱ = -0.571, p = 0.001
	genus *Enterobacter*	ϱ = -0.521, p = 0.002
Fish	order *Coriobacteriales*	ϱ = 0.511, p = 0.003
Milk and milk products	*Subdoligranulum* spp.	ϱ = 0.500, p = 0.004
Vegetable oils, plant- and animal-based fats	genus *Campylobacter*	ϱ = 0.528, p = 0.002
	genus *Fusobacterium*	ϱ = 0.528, p = 0.002
	genus *Leptotrichia*	ϱ = 0.528, p = 0.002
	genus *Porhphyromonas*	ϱ = 0.528, p = 0.002
	genus *Faecalitalea*	ϱ = 0.528, p = 0.002
	genus *Gemelia*	ϱ = 0.521, p = 0.002
	genus *Epulopiscium*	ϱ = 0.528, p = 0.002
	genus *Hungatella*	ϱ = 0.514, p = 0.003
	genus *Parvimonas*	ϱ = 0.525, p = 0.002
	family *Veillonellaceae*	ϱ = 0.529, p = 0.002
Vegetables	family *Oscillospiraceae*	ϱ = -0.542, p = 0.001
	Paludicola spp.	ϱ = 0.539, p = 0.001
Legumes, nuts, seeds, milk alternatives	*Citrobacter* spp.	ϱ = -0.518, p = 0.002
Sweets, bakery goods	*Mitsuokella* spp.	ϱ = -0.527, p = 0.002
Condiments	*Anaerotruncus* spp.	ϱ = 0.576, p = 0.001
Coffee	genus *Gastranaerophilales*	ϱ = -0.590, p < 0.0001
	genus *Victivallis*	ϱ = -0.514, p = 0.003
	genus *Anaeroplasma*	ϱ = -0.558, p = 0.001
Tea	*Coprobacter* spp.	ϱ = -0.645, p < 0.0001
Water	Family *Methanomethylophilaceae*	ϱ = -0.579, p = 0.001

Table S4. Dietary habits and gut microbiome composition related profiles based on principal component analysis.

Microorganisms at phylum level	First profile (eigenvalue = 3.05)	Second profile (eigenvalue = 2.29)	Third profile (eigenvalue = 1.43)
Actinobacteriota	**0.502**[1]	0.111	-0.231
Bacteriodota	**-0.560**	**0.534**	0.079
Firmicutes	0.451	**-0.566**	0.145
Proteobacteria	0.116	0.036	**-0.586**
Other phyla	-0.246	-0.244	0.033
**Foods products and beverages**			
Grains, grain-based products	0.136	-0.239	0.025
Bread	0.207	0.149	-0.030
Potatoes	0.059	**0.633**	0.027
Meat, offal	0.327	0.110	0.003
Meat products	0.438	0.012	0.247
Eggs	-0.083	-0.255	-0.110
Fish	0.454	-0.123	0.411
Fish products	0.355	-0.080	0.166
Milk and milk products	0.236	0.201	-0.236
Vegetable oils, plant- and ani-mal-based fats	-0.211	-0.282	-0.053
Vegetables	-0.199	**0.526**	0.317
Fruits, berries	-0.292	0.181	-0.079
Mushrooms	-0.440	-0.041	-0.127
Legumes, nuts, seeds, milk alternatives	-0.104	**-0.554**	-0.019
Sweets, bakery goods	**0.520**	0.266	-0.097
Condiments	-0.316	-0.288	0.040
Fast food, salty snacks	**0.600**	0.221	0.232
Lemonades, energy drinks	0.273	-0.056	**-0.533**
Coffee	-0.168	0.131	0.266
Tea	-0.292	-0.219	0.334
Water	-0.388	0.148	-0.136

[1] Rotated component matrix coefficients above 0.5 or less than -0.5 bolded as statistically significant values.

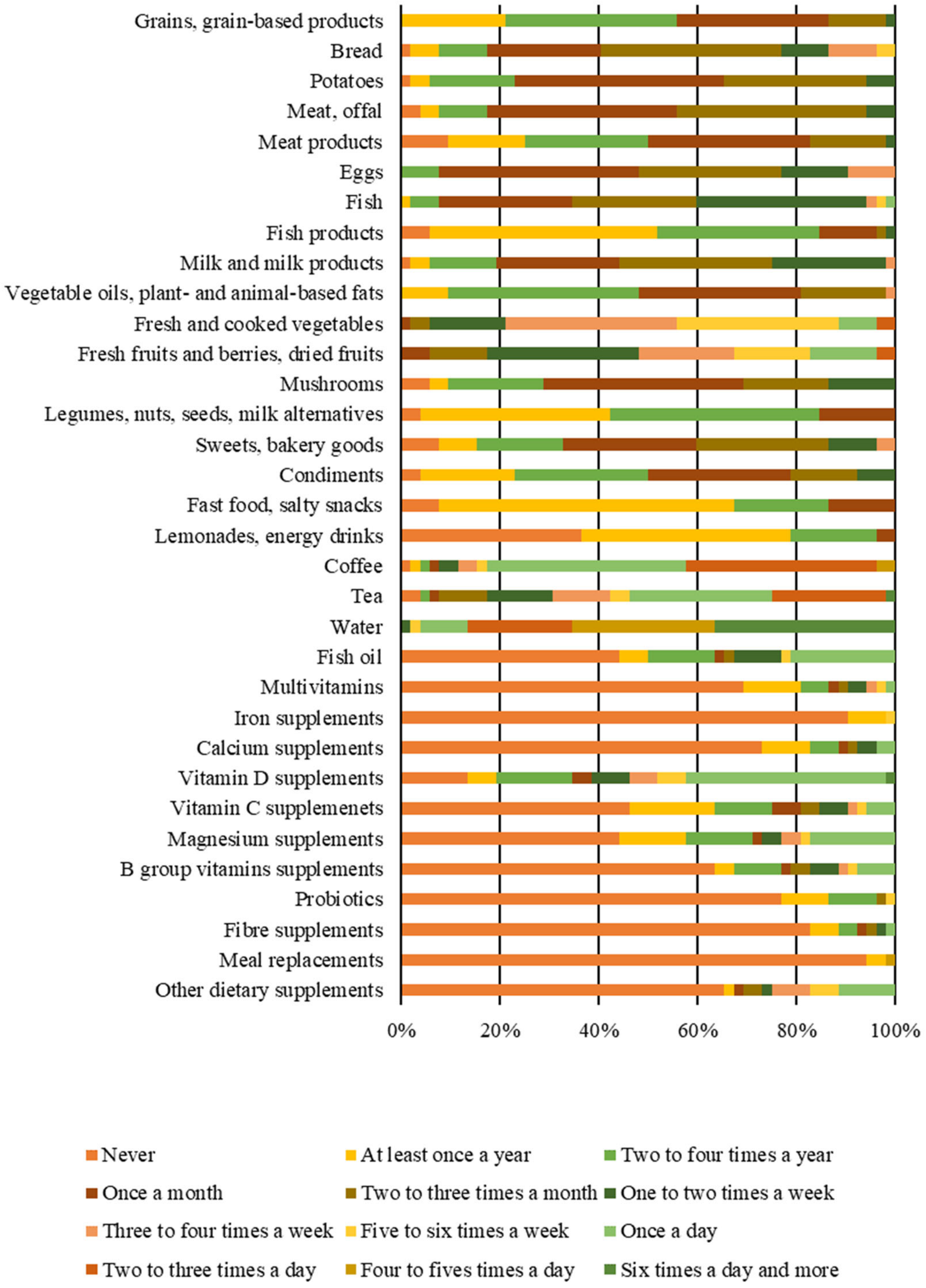

Figure S1. Average food, beverage and dietary supplements intake. Data from the food frequency questionnaire (n = 52).
